# Behavioral Risk Factors and Adherence to Preventive Measures: Evidence From the Early Stages of the COVID-19 Pandemic

**DOI:** 10.3389/fpubh.2021.674597

**Published:** 2021-06-09

**Authors:** María-José Mendoza-Jiménez, Tessa-Virginia Hannemann, Josefine Atzendorf

**Affiliations:** Munich Center for the Economics of Aging, Max Planck Institute for Social Law and Social Policy, Munich, Germany

**Keywords:** COVID-19 pandemic, preventive measures, obesity, alcohol consumption, smoking, sedentarism, unhealthy diet, ageing

## Abstract

Behavioral risk factors, such as smoking, excessive alcohol consumption, physical inactivity, obesity, and unhealthy food intake are added risk factors for severe outcomes of COVID-19 infections. Preventive measures to avoid infections are therefore particularly important for individuals engaging in behavioral risk factors. We seek to determine whether behavioral risk factors (BRFs) play a significant role in the adherence to preventive COVID-19 measures in a population aged 50 and above. The SHARE wave 8 (Survey of Health, Ageing and Retirement in Europe) and SHARE COVID-19 Survey served as the database, resulting in an analytical sample of 17,588 respondents from 23 European countries plus Israel. Of these 36.04% engaged in at least one BRF and 16.68% engaged in 3 or more BRFs. Multilevel logistic regressions revealed that engagement in one BRF was significantly associated with less adherence to hygiene preventive measures, i.e., hand-sanitizing, hand-washing and covering coughs and sneezes (OR: 0.86; 95% CI: 0.78; 0.94), as was engagement in two BRFs (OR: 0.85; 95% CI: 0.74; 0.97) and three or more BRFs (OR: 0.72; 95% CI: 0.59; 0.88). No such association was found between engagement in BRFs and adherences to social isolation preventive measures, i.e., avoiding meeting more than five people, visiting others or going shopping, or regulated preventive measures, i.e., wearing a mask and keeping physical distance. The found association was also stronger when three or more BRFs were engaged in (1 vs. 3 BRFs: χ^2^ = 3.43, *p* = 0.06; 2 vs. 3 BRFs: χ^2^ = 6.05; *p* = 0.01). The study gives insight into the protective behavior of a population with inherent vulnerability during a global health emergency. It lays the foundation for follow-up research about the evolution of adherence to preventive measures as the pandemic progresses and about long-term behavioral changes. In addition, it can aide efforts in increasing preventive compliance by raising awareness of the added risk behavioral risk factors pose.

## Introduction

The year 2020 was unequivocally marked by the emergence of the novel coronavirus and the resulting global pandemic (1), which has impacted daily life across the globe and populations. Strong recommendations have been made by health officials as well as the World Health Organization (WHO) to follow behavioral guidelines and hygiene measures: keeping distance from others, reducing contact, staying at home, washing and sanitizing hands, and wearing mouth and nose coverings (2). Although vaccinations are now underway in a number of countries, the recommended measures continue to be a central part of the pandemic response as they represent the most accessible form of preventing an infection. The present study, uses data from the Survey of Health Ageing and Retirement in Europe (SHARE) COVID-19 study (3) to investigate whether behavioral risk factors (BRFs), such as smoking and risky alcohol consumption, play a significant role in the adherence to preventive COVID-19 measures in a population aged 50 and above. As will be further discussed below, the added risk of these behaviors could lead to more protective behaviors, i.e., greater adherence to the aforementioned measures. However, given what we know of the spill over effects of risky behaviors, adherence may be lower in respondents that engage in BRFs.

While COVID-19 infections pose a potentially serious health threat to the entire population, older aged individuals and those with pre-existing medical conditions appear to be at greatest risk for severe and fatal outcomes of an infection. Public health officials and members of the medical community have also expressed serious concern about BRFs (4). A recent cohort study conducted in the UK showed a dose-dependent association between the life-style behaviors investigated in the present study and COVID-19 infections. Individuals between the ages of 40 and 69 that engaged in either smoking, elevated alcohol intake, physical inactivity, or were obese were more likely to be hospitalized due to COVID-19. The risk of hospitalization increased with the number of behaviors exhibited (5). Further, smoking has been associated with more severe symptoms as well as longer hospitalizations (6–8) and higher mortality due to COVID-19 (9). Similarly, risky alcohol consumption has been associated with adverse outcomes of a COVID-19 infection. Wang et al. (10) investigated the likelihood of contracting COVID-19 in patients with a substance use disorder diagnosis and found that individuals with an alcohol use disorder (AUD) had a greater likelihood of developing COVID-19 than individuals without substance use disorders, at an OR of 7.7. Obesity as well as associated comorbidities such as diabetes mellitus are, in addition to old age, one of the risk factors identified early on in the current pandemic. In fact, for patients treated for COVID-19, a number of studies have reported obesity as an independent factor for greater severity of infection (11, 12), longer hospitalizations (13), as well as more frequent need for ventilation (14).

Previous studies also lend insight into the link of more severe COVID-19 outcomes and BRFs by looking at their effects on immune responses in other viral infections. Nutrition has been discussed in association with COVID-19 infections, as it relates to such risk factors as obesity and diabetes mellitus (15, 16). Failing to follow healthy nutrition recommendations may therefore put individuals at a greater risk of severe outcomes from a COVID-19 infection. Regular exercise can have beneficial effects on the immune system, allowing it to better fight infections, including influenza and acute respiratory infection; failing to engage in regular physical activity, could consequently impact the body's immune responses in such a way that it poses an elevated risk for severe outcomes of an infection (17).

One can therefore accept that engagement in BRFs pose an additional risk for severe adverse outcomes of COVID-19 infections and that protective behaviors are particularly important to this population. Perceiving oneself at greater risk has been associated with greater adherence to prevention measures during the current pandemic. A study by Bíró et al. (18) found that SHARE respondents with pre-existing health conditions perceived themselves at greater risk for an infection and were also more likely to engage in prevention measures, in particular were less likely to engage in activities that involved other people, i.e., meeting people, shopping, or going out for walks. Further multinational findings highlight the role fear of COVID-19 plays in enhancing adherence to particular preventive behaviors during the current pandemic (19, 20). This is in line with most health behavior theories in which risk perception plays an important role in eliciting health behaviors in individuals (21–23). From previous pandemics, such as the SARS pandemic in 2003 and the H5N1 and H1N1 pandemic of 2012, we have learned that perceived susceptibility has had a significant effect on the use of face-masks, also beyond the pandemic (24). We may therefore assume that the elevated risk of morbidity and mortality of COVID-19 infections posed by the behaviors described above could lead to greater adherence to the preventive behaviors.

However, although these risk behaviors are often assessed in isolation, they rarely occur as such. So-called SNAP behaviors, i.e., smoking, unhealthy nutritional intake, alcohol consumption, and physical inactivity, tend to co-occur. Smoking and risky alcohol consumption have consistently been found to form an alliance of risk behaviors (25, 26), but other studies have also found all four to co-occur. They have also been found to occur with additional unhealthy habits, such as unprotected intercourse and problematic sleep patterns (27). Dolan and Galizzi (28) describe these unhealthy behaviors as promoting behavioral spill over, illustrating the propensity for taking health risks in individuals that engage in BRFs. Therefore, engaging in BRFs may be associated with greater risk taking and in the context of the current pandemic, can lead to less engagement in preventive behaviors. This was demonstrated in two Japanese studies investigating factors associated with protective behaviors during the current pandemic, finding alcohol consumption and smoking (29, 30) were associated with lower compliance with preventive COVID-19 measures. This spillover of unhealthy habits could also predict less health conscious behaviors during a global pandemic, i.e., non-compliance with protective behaviors.

Improving adherence to protective measures is one of the most important tasks during a global pandemic, especially amongst those most vulnerable to experience severe outcomes due to an infection. Yet what can lead to lesser or greater adherence is, at present, largely unknown. This study investigates whether individuals that partake in BRFs are more or less likely to participate in recommended preventive measures of hand hygiene, covering coughs and sneezes, wearing face coverings, keeping distance, avoiding meeting more than five people, avoiding going shopping, or visiting family or friends.

## Data and Methods

We used the Survey of Health, Ageing and Retirement in Europe (SHARE) as the basis for our data. SHARE is a longitudinal study of the 50+ population in 27 European countries and Israel. Additional information about the survey can be found at Börsch-Supan et al. (31) and on the project's webpage (http://www.share-project.org/organisation/share-eric.html). Furthermore, the Oxford COVID-19 Government Response Tracker (OxGRT) (32) was used for country-level data on lockdown dates, mask mandates, and number of confirmed cases per million.

### Sample and Data

The current study used the preliminary beta release 0 data of SHARE wave 8 and of the SHARE COVID-19 Survey. The SHARE wave 8 was conducted using computer-assisted personal interviews (CAPI) in panel households and for refresher samples from October 2019 until March 2020. Due to the pandemic of COVID-19 and the implementation of lockdown measures worldwide, the field phase came to an early hold in March 2020. Consequently, computer-assisted telephone interviews (CATI) were conducted (SHARE COVID-19 Survey) in panel households from June until August 2020.^1^

A total of 32,625 respondents participated in both the SHARE COVID-19 Survey and in SHARE wave 8. The final dataset included 23 European countries (Germany, Sweden, Spain, Italy, France, Denmark, Greece, Switzerland, Belgium, Czech Republic, Poland, Luxembourg, Hungary, Slovenia, Estonia, Croatia, Lithuania, Bulgaria, Cyprus, Finland, Latvia, Romania, and Slovakia) and Israel. Portugal, Malta, Austria, and the Netherlands were excluded from the original data for the purposes of the analyses. Portugal had not collected sufficient data in wave 8 to warrant inclusion. Austria's timeframe of data collection during the COVID-19 Survey differed from that of the other countries, which hinders its comparability and was thus excluded. Missing data lead to the exclusion of Malta (no country-level data in the OxGRT dataset) and the Netherlands (no data on personality measures). The sample was furthermore restricted to participants who reported leaving their home from time to time, as this stressed the need to engage in preventive measures. A sample of 29,911 respondents remained, participating in both the SHARE wave 8 and the SHARE COVID-19 Survey. Considering non-missing values on all variables considered, the final sample in our analysis consists of 17,588 individuals between the ages of 50 and 99 years.

### Measures of Variables

We first describe the variables capturing preventive behavior. Respondents in the SHARE COVID-19 Survey were asked how often they wore a facemask and kept distance when they went outside of their home. Adherence to preventive measures was considered if the responses specified that they always or often wore a facemask or kept physical distance. Non-adherence was considered when respondents indicated only sometimes or never wearing a facemask and only sometimes keeping physical distance. The respondents were also asked if they washed their hands *more* frequently than usual, if they used hand sanitizer or disinfection fluids *more* frequently than usual, or if they paid special attention to covering coughs and sneezes compared to before the pandemic. Affirmation of these questions indicated adherence to preventive measures. Furthermore, respondents were asked how often they had gone shopping, met more than five people outside of their household, or had visited other family members since the outbreak of the pandemic. For these three items, we constructed additional binary outcomes. Adherence to preventive measures was considered if the responses indicated the aforementioned behaviors had been engaged in less or had been stopped altogether since the start of the pandemic. On the contrary, if respondents answered that their behavior related to shopping, meeting more than five people outside their household, or visiting family members had increased or not changed since the start of the pandemic, non-adherence was recorded. In general, adherence to each preventive measure was coded as a binary variable taking the value of 1 when prevention—conceived as the compliance with health authorities' recommendations (2)—was undertaken.

With regard to our regressor of interest, we now describe the operationalization of the BRFs index. BRFs included habits of smoking, risky alcohol consumption, unhealthy eating habits, physical inactivity, and BMI measure from the SHARE wave 8. Smoking was assessed by asking the respondents if they were presently smoking. If yes, the variable “Presently smoking” took the value of 1. Unhealthy nutrition was defined as consuming fruits and vegetables less than daily. If yes, the variable “Unhealthy food intake” took the value of 1. Risky alcohol consumption was characterized as having consumed six or more units of alcohol on one occasion at least weekly in the last 3 months. If yes, the variable “Risky alcohol consumption” took the value of 1. Physical activity was assessed by asking the respondents how often they engaged in vigorous physical activity, such as sports, heavy housework, or a job that involves physical labor. Possible answers were *more than once a week, weekly, one to three times a month*, and *never*. The variable “Physical inactivity” took the value of 1 if the individual engaged in vigorous physical activity up to 3 times a month (i.e., answers “*one to three times a month*” or “never”), otherwise it was valued at 0 (“*weekly*” or “*more than once a week*”). The BMI was calculated based on the weight and height of the respondents and categorized into two groups: *overweight or obese* (BMI ≥ 25) or not (BMI <25). All BRFs, coded as dichotomous, were combined in an aggregate index (“BRFs index”) summing up the number of BRFs: *0 BRFs, 1 BRF, 2 BRFs*, and *3* + *BRFs*. We use the latter variable in our main analyses but provide the estimates for the disaggregated measures in the Appendix.

We obtained pandemic-related figures about the restrictions and confirmed cases of COVID-19 from the Oxford COVID-19 Government Response Tracker (OxCGRT). In order to account for the duration of the lockdown measures, we calculated the number of days since the start of the first “lockdown.” Despite the heterogeneity in the measures adopted by governments and their enforcement capacities, we considered “lockdown” as the periods for which the variable “stay at home” is at least 1, i.e., that at least recommendations to stay at home were announced. We calculated the number of days since the start of mask-wearing recommendations in a similar fashion. Both variables were calculated considering the interview date (3) and hence varied at the individual level (level 1 predictor). In the case of accumulated cases per million, the figure varied at the national level and was thus considered a level 2 predictor. The accumulated sum of cases was calculated considering cases until May 27, 2020, which coincided with the earliest interviews in the SHARE COVID-19 Survey fieldwork.

As for control variables, we consider the following. Gender, education, cohabitation, and job situation were included as sociodemographic characteristics. Education was measured by the international standard classification of education (ISCED) (33) and grouped into three categories: *0 primary, 1 secondary*, and *2 tertiary* education. To assess the status of cohabitation, a binary variable was created. One indicated that the respondent lived in the household together with their spouse or partner and *0* indicated that the respondent was either single, divorced, widowed, or lived separately from their spouse or partner. The respondents job situation was defined as *0 retired, 1 employed/self-employed*, and *2 other job situation*. Additionally, it was assessed if the respondents lived in a small town or in a rural area or if they lived in an urban area. Respondents were asked furthermore if they had chronic diseases, if they were feeling depressive, and how they would rate their health status subjectively. Depression was measured using the 12-item EURO-D scale (34). We then created a binary variable “At risk of depression” which took the value of 1 if the EURO-D measure was equal or larger than 4. Regarding the subjective health status, respondents could rate their health status as either *0 fair/poor health* or as either *1 good/very good/excellent health*. Furthermore, the 10-item Big Five inventory (35) was used to assess respondents' scores on the Big Five personality dimensions: Openness, Conscientiousness, Extraversion, Agreeableness, and Neuroticism. Each of these personality variables were transformed from an original 5-point scale with values between 1 and 5 to a 10-point scale with values between 1 and 10.

### Data Analysis Procedure

The set of outcomes we were interested in contained the eight items described previously as preventive measures. In order to reduce the dimensionality of the analysis, we ran a principal component analysis (PCA) on this set by inputting the polychoric correlation matrix to account for the ordinal nature of the variables. The three main components with eigenvalues larger than 1 were extracted, which together explain 69.83% of the variance. The items that loaded high (>0.3) on the first component were those related to going shopping, having met more than 5 people outside of the household, and having visited family members. We labeled this component “Social isolation.” In the case of the second component, which we labeled “Hygiene measures,” the higher loadings were associated with the items related to hand-washing, sanitizer use, and cough/sneeze covering. Finally, the third component “Regulated measures” was characterized mostly by mask wearing and keeping physical distance. In this study, the three components were used as outcome variables. However, given the skewness of the scores in their continuous form, they were dichotomized, taking the value of 1 if the score was larger or equal to sample's average score.

In order to examine whether adherence to preventive measures differs with engagement in BRFs in the international sample (two-level), multilevel mixed-effects logistic regressions were conducted. Specifically, we estimated the model presented in Equation (1), where *y*_*ij*_ was the binary outcome for each individual *i* in country *j*, and each β (β_1_.β_p_) corresponded to the coefficient of individual-level fixed predictors (including our predictor of interest *BRFsIndex*). γ corresponded to the coefficient of a level-2 predictor *z*, which will be explained shortly below. *u*_*j*_ were the normally-distributed country-specific random effects. Individual-specific errors are distributed as logistic and independent of *u*_*j*_. Multilevel analyses can adjust standard errors which might be biased if the hierarchical structure of the nested data is ignored in simple regression models (36, 37).

(1) $$\begin{array}{l} logit\left[\mathrm{Pr}\left(y_{ij}|x_{ij},z_{j}\right)\right]=\beta_{0}+\beta_{1}BRFsIndex_{ij}\\ +\ldots +\beta_{p}x_{pij}+\gamma z_{j}+u_{j} \end{array}$$

We started our analysis by estimating the intercept-only or null model, i.e., excluding predictors and control variables, to assess whether the adherence to preventive measures varied between countries. Second, models with fixed predictors and random intercepts were conducted, which included predictor variables at the individual level and their direct association with the outcome variables. Third, predictor variables relying on macro level information were included in the model; one of these variables, deaths per million, varied at the country-level only. The fit of the models were assessed using two goodness-of-fit measures: the Akaike Information Criterion (AIC) and the Bayesian Information Criterion (BIC). The smaller the value, the better the fit of the model.

All statistics and estimations were obtained using calibrated weights to account for unit non-response and attrition errors, which are included in the “gv_weights” module of the SHARE data.^2^ The analyses were performed using Stata 14 SE (Stata Corp LP, College Station, TX).

For requirements for a specific article type please refer to the Article Types on any Frontiers journal page. Please also refer to Author Guidelines for further information on how to organize your manuscript in the required sections or their equivalents for your field.^3^

## Results

### Summary Sample Statistics

Tables 1, 2 below report the main summary statistics of the categorical and continuous variables used in this study. The analytical sample was comprised of 17,588 individuals.^4^ In Table 1, columns 3 and 4 report the unweighted and weighted prevalence rates, respectively. In general, adherence to the three groups of preventive measures was around 60%. In terms of the BRFs, around 80% of the respondents reported engaging in at least one. We also included disaggregated behaviors. Less than half of the sample fell in the overweight or obese category (~25%), was presently smoking (~15%), ate fruits and vegetables less than daily (~25%), or displayed risky alcohol consumption (7%). Around 50% of the respondents engaged in low physical activity. Regarding the control variables, the majority of respondents were female (60.2%), and the largest age group (almost 54%) was respondents between the ages of 65–79 years old. More than half of the respondents lived with their partner or spouse. In terms of education, the majority (almost 62%) had completed a secondary level of studies. Twenty percentage of the sample reported to be employed or self-employed; 69% as retired. Most respondents did not live in urban locations (almost 60%). In terms of health, 32.6% were screened as at risk for depression, whereas a large majority reports at least good self-perceived health (~73%). As can be read from an overall comparison of weighted and unweighted figures, the sample composition and the population's composition are not considerably different.

**Table 1:** Descriptive statistics of the analytical sample for categorical variables.

	**Frequency in sample**	**Prevalence (%, unweighted)**	**Prevalence (%, weighted)**
**Outcomes**			
*Engagement in…*			
Social isolation measures	10,570	60.10	57.47
Hygiene measures	12,117	68.89	70.31
Regulated measures	11,586	65.87	65.69
**Regressors**			
No. of BRFs			
0 (reference cat.)	3,090	17.57	17.58
1	6,384	36.30	36.04
2	5,368	30.52	29.70
3 or more	2,746	15.61	16.68
BMI ≥ 25	4,334	24.64	25.12
Presently smoking	2,666	15.16	18.26
Unhealthy food intake	4,352	24.74	26.37
Physical inactivity	8,945	50.86	48.07
Risky alcohol consumption	1,147	6.52	7.06
Sex, male (*Ref*.)	7,000	39.80	42.55
Age			
50–55 y/o	714	4.06	8.70
56–64 y/o (*Ref*.)	5,136	29.20	41.39
65–79 y/o	9,465	53.82	39.35
80+ y/o	2,273	12.92	10.55
Spouse/partner living in HH	11,136	63.32	56.97
Education			
Primary (*Ref*.)	2,122	12.07	9.98
Secondary	10,886	61.89	63.41
Tertiary or above	4,580	26.04	26.61
Employment			
Retired	12,140	69.02	54.05
Employed/self-employed	3,598	20.46	33.13
Other	1,850	10.52	12.82
Living in an urban environment	7,267	41.32	40.13
Depression risk (EURO-D)	5,734	32.60	32.66
Subjective health			
Fair/poor (*Ref*.)	4,738	26.94	24.78
At least good	12,850	73.06	75.22
*n*	17,588		

*Unless otherwise indicated, the non-endorsement of described behavior was used as the reference category. Data: Wave 8 Release 0.0.1 beta*.

**Table 2:** Descriptive statistics of the analytical sample for continuous variables.

	**Mean (Unw.)**	**SD (Unw.)**	**Mean (Wgt.)**	**SD (Wgt.)**	**Min**	**Max**
**Regressors**						
No. of chronic diseases	1.26	1.21	1.15	1.19	0	8
*Big 5 personality traits*					
Openness	6.72	1.89	6.73	1.87	2	10
Conscientiousness	8.26	1.58	8.26	1.58	2	10
Extraversion	7.04	1.85	7.04	1.85	2	10
Agreeableness	7.42	1.64	7.41	1.64	2	10
Neuroticism	5.23	2.03	5.23	2.03	2	10
Days in LD since first LD	84.27	22.65	84.35	22.95	49	157
Days since start of masks enforcement	63.21	44.30	63.87	43.99	0	191
Cases per million	1,956.28	1,572.95	1,957.67	1,574.82	270.03	6,380.69

*Unweighted (Unw.) and weighted (Wgt.) average and standard deviation (SD) are presented. Data: Wave 8 Release 0.0.1 beta*.

With respect to the variables we treated as continuous, Table 2 reports figures for mean and standard deviation (SD). On average, respondents reported one chronic disease. Finally, regarding the information related to the pandemic, on average, interviews took place after approximately 84 days under “lockdown” and around 60 days with mask-wearing recommendations or regulations. Weighted figures were not considerably different.

Considering the country-level prevalence of each preventive measure, Tables A1, A2 in the Appendix report an overall high adherence to the measures (above 65%). The lowest adherence to mask-wearing was found in Sweden (~2%), Finland (~5%) and Denmark (~3%). Compared to mask-wearing, overall adherence to keeping physical distance (~95%), washing hands (~90%), using hand sanitizer (~85%) and covering cough/sneezes (~85%) seemed higher. Regarding social isolation behaviors (Table A2 in the Appendix), most of the participants reported having engaged in these behaviors “less often,” specifically for “having met five people” (~87%) and for “visited people” (~83%). In the case of “went shopping,” more respondents reported that their engagement in this activity increased or remained unchanged, however, the “less often” category still contains most of the sample (~69%) in most countries, except for Denmark (~49%), Bulgaria (~47%) and Slovakia (~45%).

Table A3 in the Appendix shows the country-level prevalence of each behavioral risk. The highest prevalence rates for being overweight or obese could be found in Latvia (~38%), Hungary (~33%), and Estonia (~33%). Switzerland (~14%), Italy (~15%) and Israel (~18%) had the lowest prevalence rates of being overweight and obese in the sample. Approximately one-quarter of the sample reported that they were presently smoking in Croatia (~25%) and Poland (~24%). Sweden (~6%) and Finland (~10%) were the countries with the lowest prevalence rate of smokers. In Bulgaria (~66%), Romania (~55%), and Slovakia (~58%), more than half of the respondents displayed unhealthy eating habits, whereas in Luxembourg (~13%), France (~10%), and Slovenia (~13%) about one-tenth of the sample reported unhealthy eating habits. In Hungary (~64%), Cyprus (~64%) and Spain (~64%), the majority of the sample were physically inactive. The lowest prevalence rates for physical inactivity were found in Finland (~33%), Denmark (~36%), and Latvia (~39%). Risky alcohol consumption was highest in Cyprus (~21%) and Bulgaria (~16%) and lowest in France (~3%), Poland (~2%) and Israel (~2%). Table A3 also presents the equivalent rates using calibrated weights, which did not differ considerably.

### Results on Social Isolation

As shown in Table 3, the variance component for the intercept-only model of the social isolation component was statistically significant, indicating that the adherence to social isolation measures differed between countries (AIC = 23,210.96, BIC = 23,226.51). The ICC revealed that 9% of the variance of the social isolation component could be explained by variations between the countries.

**Table 3:** Multilevel analyses predicting adherence to social isolation (*n* = 17.588), Data: Wave 8 Release 0.0.1 beta.

	**(1)**		**(2)**		**(3)**	
	**Intercept-only model**		**Fixed predictors at individual level with random intercepts**		**Fixed predictors at individual and macro level with random intercepts**	
**Regressors**	**OR**	**CI (95%)**	**OR**	**CI (95%)**	**OR**	**CI (95%)**
Intercept	1.50^**^	(1.05, 2.15)	1.00	(0.60, 1.67)	0.82	(0.28, 2.40)
BRFs						
0 BRF (*Ref*.)						
1 BRF			1.09	(0.94, 1.27)	1.10	(0.94, 1.28)
2 BRFs			1.01	(0.82, 1.25)	1.02	(0.83, 1.25)
3+ BRFs			1.06	(0.95, 1.18)	1.06	(0.95, 1.19)
Sex						
Male (*Ref*.)						
Female			1.74^***^	(1.32, 2.29)	1.74^***^	(1.32, 2.29)
Age						
50–55 y/o			1.02	(0.72, 1.45)	1.02	(0.72, 1.45)
56–64 y/o (*Ref*.)						
65–79 y/o			1.25^**^	(1.00, 1.56)	1.25^**^	(1.00, 1.55)
Older than 80 y/o			1.50^***^	(1.22, 1.85)	1.50^***^	(1.22, 1.83)
Living situation						
Living alone (*Ref*.)						
Spouse/partner in HH			1.47^***^	(1.13, 1.91)	1.47^***^	(1.14, 1.90)
Education						
Primary (*Ref*.)						
Secondary			1.02	(0.95, 1.10)	1.02	(0.94, 1.10)
Tertiary or above			1.11	(0.92, 1.33)	1.11	(0.92, 1.33)
Employment						
Retired (*Ref*.)						
Employed/self-employed			0.91	(0.69, 1.21)	0.91	(0.69, 1.21)
Other			0.97	(0.83, 1.15)	0.97	(0.83, 1.14)
Living environment						
Rural (*Ref*.)						
Urban			0.94	(0.81, 1.10)	0.94	(0.81, 1.09)
Depression risk (EURO-D)			1.04	(0.95, 1.12)	1.04	(0.96, 1.12)
Subjective health						
Fair/poor (*Ref*.)						
At least good			0.58^***^	(0.44, 0.77)	0.59^***^	(0.44, 0.78)
No. of chronic diseases			1.04	(0.96, 1.13)	1.04	(0.96, 1.13)
Big five personality traits						
Openness			1.01	(0.98, 1.03)	1.01	(0.98, 1.03)
Conscientiousness			0.99	(0.95, 1.03)	0.99	(0.95, 1.03)
Extraversion			0.98^*^	(0.95, 1.00)	0.98^*^	(0.95, 1.00)
Agreeableness			1.01	(0.97, 1.05)	1.01	(0.97, 1.05)
Neuroticism			1.04^**^	(1.00, 1.08)	1.04^**^	(1.01, 1.08)
Days in LD^a^ since first LD					1.00	(0.99, 1.01)
Days since start of mask enforcement before int.					1.00	(0.99, 1.00)
Cases per million					1.00^***^	(1.00, 1.00)
	**Var**.	**CI (95%)**	**Var**.	**CI (95%)**	**Var**.	**CI (95%)**
Intercept	0.32^***^	(0.18, 0.55)	0.34^***^	(0.20, 0.57)	0.25^***^	(0.10, 0.62)
ICC	0.09		0.09		0.07	
BIC	23,226.51		22,601.45		22,601.28	
AIC	23,210.96		22,422.62		22,414.68	

*OR, Odds ratios; CI (95%), confidence interval 95%; Var., variance component*;

*** *p < 0.01*,

** *p < 0.05*,

* *p < 0.1*.

a *LD stands for “lockdown”*.

**Figure 1:**
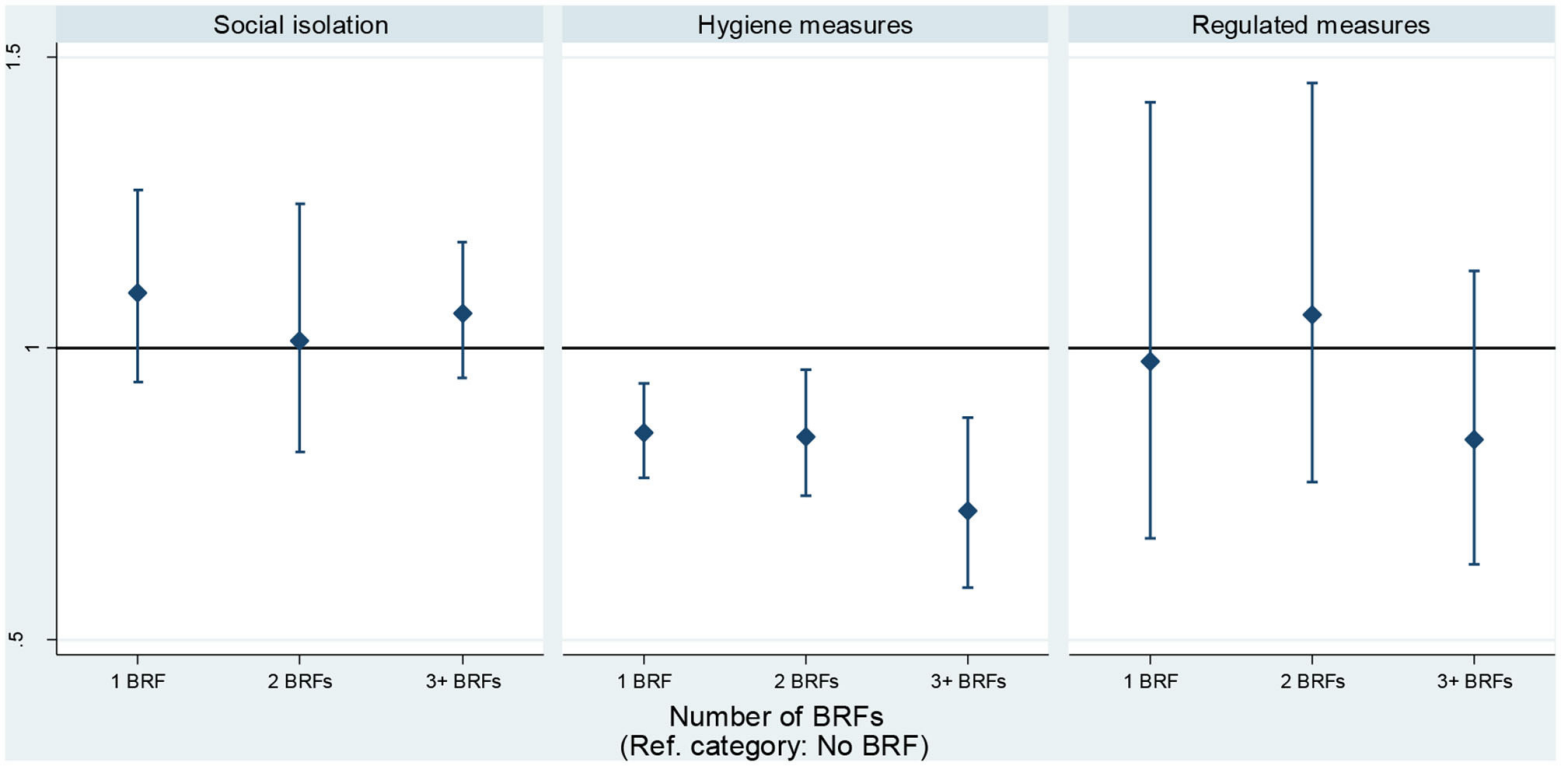
Association between behavioral risk factors and preventive measures. Estimates based on mixed-effects logistic regressions, controlling for socio-demographic and health variables collected before the start of the pandemic (*n* = 17,588). Data: Wave 8 Release 0.0.1 beta.

At the individual level, the second model in Table 3 shows there was no significant association between the social isolation component and the BRFs index. This result can also be graphically inspected in the left panel of Figure 1. At the macro level, the number of cases of infection (OR = 1.0002; 95% CI: 1.0001–1.0003) were significantly associated with higher adherence to preventive measures regarding social isolation. When including variables at the individual and macro level, the model fit improved compared with the intercept-only model (AIC = 22,414.68; BIC = 22,601.28).

When the BRFs were included individually (Table A4), smoking (OR = 0.73; 95% CI: 0.62–0.86) and unhealthy eating habits (OR = 1.14; 95% CI: 1.05–1.25) were associated with lower and higher adherence to social isolation measures, respectively. Table A5 presents the estimates for the disaggregated BRFs using as outcomes each of the preventive measures associated to social isolation (columns 6–8).

### Results on Hygiene Measures

Table 4 reports the estimates for the second outcome, adherence to hygiene measures. The variance component for the intercept-only model was statistically significant, indicating that the adherence to hygiene measures differed between countries (AIC = 21,033.30, BIC = 21,048.85). The ICC revealed that 6% of the variance of this outcome could be explained by variations between the countries.

**Table 4:** Multilevel analyses predicting adherence to hygiene measures (*n* = 17.588), Data: Wave 8 Release 0.0.1 beta.

	**(1)**		**(2)**		**(3)**	
	**Intercept-only model**		**Fixed predictors at individual level with random intercepts**		**Fixed predictors at individual and macro level with random intercepts**	
**Regressors**	**OR**	**CI (95%)**	**OR**	**CI (95%)**	**OR**	**CI (95%)**
Intercept	2.69^***^	(1.96, 3.68)	0.74	(0.46, 1.20)	0.12^***^	(0.04, 0.35)
BRFs						
0 BRF (*Ref*.)						
1 BRF			0.85^***^	(0.78, 0.94)	0.86^***^	(0.78, 0.94)
2 BRFs			0.85^**^	(0.75, 0.96)	0.85^**^	(0.74, 0.97)
3+ BRFs			0.72^***^	(0.59, 0.88)	0.72^***^	(0.59, 0.88)
Sex						
Male (*Ref*.)						
Female			1.39^***^	(1.29, 1.50)	1.40^***^	(1.29, 1.51)
Age						
50–55 y/o			1.32^***^	(1.07, 1.63)	1.34^***^	(1.08, 1.65)
56–64 y/o (*Ref*.)						
65–79 y/o			0.88	(0.75, 1.03)	0.88	(0.75, 1.04)
Older than 80 y/o			0.66^***^	(0.54, 0.80)	0.66^***^	(0.54, 0.81)
Living situation						
Living alone (*Ref*.)						
Spouse/partner in HH			1.47^***^	(1.36, 1.59)	1.46^***^	(1.35, 1.58)
Education						
Primary (*Ref*.)						
Secondary			1.26^**^	(1.02, 1.54)	1.28^**^	(1.03, 1.60)
Tertiary or above			1.72^***^	(1.49, 2.00)	1.76^***^	(1.50, 2.06)
Employment						
Retired (*Ref*.)						
Employed/self-employed			1.27^**^	(1.03, 1.57)	1.26^**^	(1.02, 1.55)
Other			1.11	(0.85, 1.45)	1.10	(0.84, 1.43)
Living environment						
Rural (*Ref*.)						
Urban			1.06	(0.86, 1.31)	1.07	(0.86, 1.33)
Depression risk (EURO-D)			0.96	(0.87, 1.06)	0.96	(0.87, 1.06)
Subjective health						
Fair/poor (*Ref*.)						
At least good			1.01	(0.90, 1.12)	1.00	(0.90, 1.11)
No. of chronic diseases			1.07^**^	(1.00, 1.15)	1.08^**^	(1.01, 1.15)
Big five personality traits						
Openness			0.98^**^	(0.96, 1.00)	0.98^***^	(0.96, 1.00)
Conscientiousness			1.05^*^	(1.00, 1.09)	1.05^*^	(1.01, 1.09)
Extraversion			0.96^***^	(0.94, 0.99)	0.96^***^	(0.94, 0.99)
Agreeableness			1.08^***^	(1.06, 1.11)	1.08^***^	(1.06, 1.11)
Neuroticism			1.03^**^	(1.00, 1.06)	1.03^**^	(1.00, 1.05)
Days in LD^a^ since first LD					1.01^**^	(1.00, 1.02)
Days since start of mask enforcement before int.					1.00^*^	(1.00, 1.01)
Cases per million					1.00^*^	(1.00, 1.00)
	**Var**.	**CI (95%)**	**Var**.	**CI (95%)**	**Var**.	**CI (95%)**
Intercept	0.21^***^	(0.12, 0.38)	0.23^***^	(0.13, 0.41)	0.21^**^	(0.09, 0.48)
ICC	0.06		0.07		0.06	
BIC	21,048.85		20,592.02		20,545.39	
AIC	21,033.30		20,413.20		20,366.57	

*OR, Odds ratios; CI (95%), confidence interval 95%; Var., variance component*;

*** *p < 0.01*,

** *p < 0.05*,

* *p < 0.1*.

a *LD stands for “lockdown”*.

When including the set of individual-level (Level 1) controls, respondents with at least one behavioral risk factor were associated with lower adherence to preventive hygiene measures compared to respondents who did not engage in any of them [1 BRF: OR = 0.86; 95%-CI = (0.78; 0.94), 2 BRF: OR = 0.85; 95%-CI = (0.74; 0.97), 3+ BRF: OR = 0.72; 95%-CI = (0.59; 0.88)]. These estimates decreased as the number of BRFs increases. The middle panel in Figure 1 illustrates the relationship. We tested for significant differences between the coefficients of these categories. The Wald test results indicate that the coefficient for the category “2 BRFs” is not significantly different than the coefficient of the category “1 BRF” (χ^2^ = 0.01, *p* = 0.90). However, the difference between the coefficient for “3 or more BRFs” and that of “1 BRF” was significant (χ^2^ = 3.43, *p* = 0.06), as was the difference between the coefficient for “3 or more BRFs” and “2 BRFs” (χ^2^ = 6.05; *p* = 0.01).

At the macro level, the last two columns in Table 4 show the number of days in lockdown (OR = 1.01; 95% CI: 1.00–1.02) was associated with higher adherence to preventive measures regarding hygiene. In the case of the number of days with mask-wearing regulations (OR = 1.005; 95% CI: 0.99–1.01) and cases per million (OR = 1.000; 95% CI: 0.99–1.00), the results report a direct association significant at an alpha-level of 0.1. The model with fixed predictors and random intercepts at individual and macro levels improved compared with the intercept-only model (AIC = 20,366.57; BIC = 20,545.39).

When the BRFs were included individually, smoking (OR = 0.87; 95% CI: 0.79–0.97) showed a negative association with the hygiene component (Table A4 in the Appendix). Table A5 in the Appendix presents the estimates for the disaggregated BRFs using as outcomes each of the preventive measures associated to hygiene (columns 3–5).

### Results on Regulated Measures

As shown in Table 5, the variance component for the intercept-only model of the regulated measures outcome was statistically significant, indicating that the adherence to regulated measures differed between countries (AIC = 11,584.59, BIC = 11,600.14). The ICC revealed that 48% of the variance of adherence to regulated measures could be explained by variations between the countries.

**Table 5:** Multilevel analyses predicting adherence to regulated measures (*n* = 17.588), Data: Wave 8 Release 0.0.1 beta.

	**(1)**		**(2)**		**(3)**	
	**Intercept-only model**		**Fixed predictors at individual level with random intercepts**		**Fixed predictors at individual and macro level with random intercepts**	
**Regressors**	**OR**	**CI (95%)**	**OR**	**CI (95%)**	**OR**	**CI (95%)**
Intercept	6.76^***^	(3.28, 13.90)	0.58	(0.11, 2.96)	0.05^***^	(0.01, 0.49)
BRFs						
0 BRF (*Ref*.)						
1 BRF			0.98	(0.67, 1.42)	0.98	(0.67, 1.43)
2 BRFs			1.06	(0.77, 1.46)	1.06	(0.76, 1.46)
3+ BRFs			0.84	(0.63, 1.13)	0.85	(0.63, 1.15)
Sex						
Male (*Ref*.)						
Female			1.57^***^	(1.35, 1.84)	1.58^***^	(1.35, 1.85)
Age						
50–55 y/o			1.51^***^	(1.17, 1.95)	1.51^***^	(1.16, 1.97)
56–64 y/o (*Ref*.)						
65–79 y/o			1.01	(0.66, 1.54)	1.02	(0.67, 1.55)
Older than 80 y/o			0.68	(0.43, 1.08)	0.69	(0.43, 1.09)
Living situation						
Living alone (*Ref*.)						
Spouse/partner in HH			1.20	(0.96, 1.51)	1.19	(0.95, 1.49)
Education						
Primary (*Ref*.)						
Secondary			1.15	(0.85, 1.57)	1.17	(0.88, 1.55)
Tertiary or above			1.47	(0.91, 2.40)	1.49^*^	(0.94, 2.37)
Employment						
Retired (*Ref*.)						
Employed/self-employed			0.82	(0.59, 1.14)	0.81	(0.58, 1.14)
Other			0.87	(0.73, 1.03)	0.86^*^	(0.72, 1.02)
Living environment						
Rural (*Ref*.)						
Urban			1.20	(0.94, 1.53)	1.21	(0.95, 1.54)
Depression risk (EURO-D)			1.01	(0.89, 1.15)	1.01	(0.89, 1.15)
Subjective health						
Fair/poor (*Ref*.)						
At least good			1.13	(0.87, 1.47)	1.12	(0.87, 1.44)
No. of chronic diseases			1.18^***^	(1.04, 1.34)	1.18^***^	(1.04, 1.34)
Big five personality traits						
Openness			1.05^**^	(1.01, 1.10)	1.05^**^	(1.01, 1.10)
Conscientiousness			1.15^***^	(1.07, 1.25)	1.15^***^	(1.07, 1.25)
Extraversion			0.95^***^	(0.91, 0.98)	0.95^***^	(0.91, 0.98)
Agreeableness			1.05	(0.98, 1.12)	1.05	(0.98, 1.13)
Neuroticism			1.04^**^	(1.01, 1.08)	1.04^**^	(1.00, 1.08)
Days in LD^a^ since first LD					1.02^**^	(1.00, 1.04)
Days since start of mask enforcement before int.					1.01^**^	(1.00, 1.01)
Cases per million					1.00	(1.00, 1.00)
	**Var**.	**CI (95%)**	**Var**.	**CI (95%)**	**Var**.	**CI (95%)**
Intercept	3.00^**^	(1.21, 7.45)	3.22^**^	(1.31, 7.92)	3.04^**^	(1.35, 6.83)
ICC	0.48		0.49		0.48	
BIC	11,600.14		11,445.72		11,421.97	
AIC	11,584.59		11,266.90		11,243.15	

*OR, Odds ratios; CI (95%), confidence interval 95%; Var., variance component*;

*** *p < 0.01*,

** *p < 0.05*,

* *p < 0.1*.

a *LD stands for “lockdown”*.

At the individual level, there was no significant association between the regulated measures component and the BRFs index. At the macro level, the last two columns in Table 5 report that the number of days under lockdown (OR = 1.02; 95% CI: 1.00–1.04) and the number of days with mask-wearing enforcement (OR = 1.01; 95% CI: 1.00–1.01) were both significantly associated with higher adherence to regulated measures. The model with fixed predictors and random intercepts at individual and macro levels improved compared with the intercept-only model (AIC = 11,243.15; BIC = 11,421.97).

When the BRFs were included individually and not combined as an index, Table A4 in the Appendix shows that unhealthy eating (OR = 0.75; 95% CI: 0.56–0.99) was associated with lower adherence to regulated measures. Table A5 in the Appendix presents the estimates for the disaggregated BRFs using as outcomes mask-wearing and keeping physical distance (columns 1 and 2).

### Robustness Tests

In Table A6 in the Appendix we provide estimates for alternative models using the same outcome variables as in Tables 3–5. Panel A presents an overview of the results described above, displaying the estimates for the BRFs index only. Panel B presents the equivalent estimates for Equation (1) without weights, whereas Panels C and D report the estimates from logistic regression with country fixed effects and a multi-level linear regression, respectively. In general, the results do not vary across models. When testing the differences between the coefficients (“2 BRFs vs. 3+ BRFs” and “1 BRF vs. 3+ BRFs”), these are significant at alpha-levels of 0.05 and 0.1 throughout Panels A, B, and D. The robustness tests show equivalent findings across tested models.

## Discussion

The present study provides important insight into preventive behaviors of some of the most vulnerable populations during a global pandemic. Due to their age alone, the respondents are at high risk for morbidity and mortality resulting from a COVID-19 infection. The additional engagement in behavioral risks, such as smoking, risky alcohol consumption, unhealthy eating habits, physical inactivity, and obesity, elevates the risk even further. In this context, unveiling differences in adherence to the recommended prevention measures is vital to improving compliance and reducing their risk of contracting the virus in the first place.

In general, adherence to prevention is in line with the array of empirical findings in other countries studying the pandemic. For all three prevention outcomes, women, the more educated, and those dealing with more chronic diseases report higher adherence to preventive behavior (38, 39). Regarding our research question, the results show a significant association between behavioral risk factors and prevention related to hygiene measures, as people engaged in at least one BRF were less likely to wash their hands, sanitize their hands, or cover coughs and sneezes more often than before the outbreak of the pandemic. Interestingly, other behaviors such as keeping distance and mask-wearing, as well as reducing contact to others were not related to engagement in BRFs.

Conversely to what may have been expected, individuals with BRFs did not report higher adherence to regulated measures or social isolation recommendations, despite their enhanced vulnerability for severe outcomes of a COVID-19 infection. A mechanism that might have explained higher adherence to said measures could have been higher self-perceived risk to an infection. A review study by Sim et al. (24) used the Health Belief Model (21), an established theory on health behavior, to explain influences on face-mask wearing behavior in response to the previous SARS pandemic. The study found that perceived susceptibility to an infection lead to a greater likelihood of face-mask wearing. In the context of this pandemic, empirical studies have already confirmed that greater perception of personal risk or fear of the virus resulted in a greater likelihood of engaging in preventive measures across a number of countries (39–42). This influence was not reflected in our findings. Respondents that engaged in at least one BRF were not more likely to adhere to these preventive measures than respondents who did not. Interestingly, in an analysis of the SHARE Corona Survey, Bíró et al. (18) found that respondents with pre-existing health conditions were more likely to adhere to preventive measures, particularly those involving others, i.e., going shopping, meeting with others and taking walks. This shows, that an awareness of one's susceptibility is important for its effect on protective behaviors among those with concurrent medical conditions. In this sense, awareness of the increased risk of morbidity and mortality of COVID-19 infections posed by the BRFs is lacking. Due to their added stress on immune responses and overall health, BRFs can be considered pre-existing conditions as well, even if their effects may not yet have manifested in disease.

In contrast to the hygiene preventive behaviors, which are fully voluntary protective measures, regulations have been formed around mask-wearing, social and physical distancing. In the case of social distancing measures, many countries had restrictions on the amount of people allowed to meet, as well as shops' operations or opening hours. Together, implementations of this kind rendered the preventive measure of avoiding shopping or limiting social meetings non-voluntary. Similarly, reminders to physically distance are present in a number of locations, or are enforced otherwise, e.g., by only allowing a certain number of people. Interestingly the study by Sim et al. (24) also found face-mask wearing was associated with so-called cues to action, i.e., environmental factors that influence one's choice to engage in the preventive behavior. Regulations around daily life, as seen in the pandemic response can be considered environmental cues to action. Mandatory mask-wearing had been implemented in the majority of European countries (32) included in this study and is a strong predictor for their use (43). Consequently, non-adherence can result in fines of up to €1,000 (44), illustrating a motivator that cannot be readily applied to preventive hygiene. In addition, mask mandates apply to a number of public places, such as shops, restaurants, or public transport, and entry to these places can be denied if a mask is not worn, illustrating further social repercussions of non-adherence into locales or only occupying every other table in restaurants. Regulatory measures are, however, not the only environmental factors that may influence protective behaviors. How likely the virus is to be transmitted in one's environment (45) may have an impact, as well as social norms. Non-compliance with social isolation measures, such as meeting with and visiting others, as well as standing very close to others, also relies on the adherence (or in fact non-adherence) of the other persons' tolerance of such behavior and is therefore influenced by outside, environmental factors. A study by Barceló and Sheen (46) showed that the initiation of voluntary mask-wearing was most likely in places in which mask-wearing was already popular, thus highlighting the importance of social-norms in this particular preventive behavior. This is also in line with our finding that the longer mask regulations had been in place, the greater the likelihood that respondents adhered to this measure. Put simply, several external factors seem to be underlying our findings with respect to no association between BRFs and regulated or social distancing measures, which include law and socially-driven enforcement.

The findings in the study also demonstrate that engagement in multiple BRFs increases the likelihood of non-compliance with preventive hygiene measures. This is in line with previous studies, which confirm BRFs do not only co-occur but are also associated with other health risk behaviors, including drunk driving, intoxicated or unprotected sexual intercourse, less hours of sleep, and, of course, the misuse of substances other than nicotine and alcohol (27). Not only does this confirm that non-compliance with preventive behaviors also constitutes unhealthy habits effected by this risk spillover, it also suggests that these behaviors pertain to a particular at risk population.

In order to evaluate whether pandemic-specific factors mediated the association between BRFs and preventive measures, we included the following variables in the model: national-level cases per million and duration of regulated measures (lockdown and masks) until the interview date. Lower adherence to preventive measures over time since the start of the pandemic has been evidenced in different countries, such as the United Kingdom (47) and Brazil (48). The underlying mechanisms have been attributed to multiple factors, including worsened life circumstances and lack of updated official information, not simply lower motivation. In this study, higher adherence to hygiene and regulated measures over time suggest a different scenario in the older-age population of European countries. This may reflect the extended financial assistance provided during the pandemic, but also the improved enforcement level, as pecuniary penalties were eventually introduced. In the cases of confirmed cases per million, a high number of infection rates may illicit anxiety or greater threat perception. Given the established link between perceived risk and prevention, this may provide an explanation for the increase in adherence to preventive measures with the number of cases of infections within a country. Importantly, though, the fact that the estimates for the BRFs index change only minimally when these variables are introduced suggest that the association between BRFs and preventive hygiene measures is not mediated by pandemic-related factors.

The study lends important insights to benefit public health endeavors addressing the response of individuals to the threats of the current and possibly future pandemic, particularly considering the adherence to recommended prevention of viral transmissions. An important determinant of this could be to increase the awareness of the risks posed by BRFs through effective risk communication. Heydari et al. (49) found that risk communication had a significant effect on preventive behaviors against COVID-19 infections in an Iranian sample both directly and indirectly by increasing risk perception, which in turn improved protective behaviors. In addition, the effectiveness of such risk communication is improved when the messages are tailored to target audiences (50, 51). Our findings can also support tailoring efforts by drawing a clearer picture of the population at hand, i.e., individuals aged 50 and above, engaging in multiple BRFs, as well as the protective behavior least likely observed, i.e., hand-washing, hand sanitizing and covering coughs and sneezes. Further knowledge on the possible influences of the combination of BRFs on protective behaviors can provide more details that can be utilized in tailoring communications.

Some considerations need to be taken into account. The information on the preventive behaviors was gathered in the summer of 2020. Therefore the findings can be considered a snapshot of early behavioral responses to the outbreak of the pandemic. In particular, it should be considered that most countries were more severely hit during subsequent waves, with rates of confirmed cases and deaths surpassing those registered in April-May 2020, as well as possible fatigue of implemented measures. Insights into how behavior evolved together with the pandemic can be achieved with follow-up studies using the advantage of the panel characteristic of the SHARE survey. Second, our analysis is based on behaviors that are subject to social desirability bias. We argue that the presence of such bias would work in opposite directions (underreporting behavioral risk factors and over-reporting preventive measures), potentially masking a statistically significant association. In our case, the estimated association would be underestimated. Third, the efforts to feasibly collect cross-country data from a harmonized questionnaire relied on the change in mode between the SHARE w8 (CAPI) interview and the SHARE COVID-19 (CATI) survey. This adaptation could have limited the participation of potential respondents and thus the sample composition in a non-random fashion. However, the mode change was implemented in the same way in all SHARE countries.

We have provided estimates using data from the SHARE project, a multinational panel study, which has a number of advantages. The sample is representative and potential underrepresentation derived from non-response has been addressed by including calibrated weights. In addition, we are able to use the panel dimension to our study's advantage. As our measures for BRFs and prevention were collected at different points in time, we were able to capture the BRFs outside of the context of the pandemic, reflecting much more typical behavior in our sample. In a similar vein, the panel dimension and harmonization of the questionnaire across countries sets the ground for follow-up studies interested in the characterization of the found associations across time. Particularly in this regard, the SHARE Corona Survey 2, planned to be fielded in the summer of 2021, will provide additional input for contrasting our results in a more advanced stage of the pandemic.

## Conclusion

Our findings suggest that BRFs play a part in the engagement of voluntary protective measures, i.e., hygiene measures, and that this is more pronounced in individuals that engage in multiple BRFs. This characterization of preventive behavior is a snapshot of an immediate response to this global health emergency and should be further monitored with the continuation of the pandemic. The contributions of our study are at least three-fold. First, we add to the growing array of works documenting the short-run response to the pandemic, a health emergency that is still evolving as these lines are written. Second, our findings emphasize health risks among a highly vulnerable population. Third, building on previous health studies that have reported the co-occurrence of behavioral risk factors, we expand this understanding by analyzing these behaviors in tandem. It therefore highlights the importance of promoting preventive behaviors amongst a high-risk population. The study can be used as a foundation for tailored risk communication and used by stakeholders in public health to address the role that BRFs play in the vulnerability for morbidity and mortality due to a COVID-19 infection, especially amongst older individuals.

## Data Availability Statement

Publicly available datasets were analyzed in this study. All data used in our study are available free of charge to all scientific users world-wide after individual registration (http://www.share-project.org/data-access/user-registration.html). SHARE data are DOI registered datasets (http://www.share-project.org/data-documentation/share-data-releases.html). Each wave and each release is assigned a persistent DOI. In our article we use SHARE data from Waves 1, 2, 3, 4, 5, 6, 7, and 8 (DOIs: 10.6103/SHARE.w1.710, 10.6103/SHARE.w2.710, 10.6103/SHARE.w3.710, 10.6103/SHARE.w4.710, 10.6103/SHARE.w5.710, 10.6103/SHARE.w6.710, 10.6103/SHARE.w7.711, 10.6103/SHARE.wXcvr.710, 10.6103/SHARE.w8cabeta.001) that are fully available without restrictions.

## Ethics Statement

The SHARE study is subject to continuous ethics review. During Waves 1 to 4, SHARE was reviewed and approved by the Ethics Committee of the University of Mannheim. Wave 4 of SHARE and the continuation of the project were reviewed and approved by the Ethics Council of the Max Planck Society. For more details please see: http://www.shareproject.org/fileadmin/pdf_documentation/MPG_Ethics_Council_SHARE_overall_approval_29.05.2020__en_.pdf. The patients/participants provided their written informed consent to participate in this study.

## Author Contributions

M-JM-J, T-VH, and JA contributed equally to the conceptualization, methodological considerations and implications. T-VH conducted the theoretical considerations. M-JM-J and JA conducted data curation as well as analyses. All authors contributed to the writing of the paper and have read and approved the final manuscript.

## Conflict of Interest

The authors declare that the research was conducted in the absence of any commercial or financial relationships that could be construed as a potential conflict of interest.

## Footnotes

^1^The paper version of this questionnaire is available online.

^2^For detailed information about the weights and the weighting strategy, please refer to the SHARE COVID-19 Survey Release Guide and the SHARE Release Guide 7.1.1 (section 15), available online.

^3^For Original Research articles, please note that the Material and Methods section can be placed in any of the following ways: before Results, before Discussion or after Discussion.

^4^Sample size figures for each country are presented in the second column of Tables A1–A3 in the Appendix.
