# Supplementary material for: Behavioral Risk Factors and Adherence to Preventive Measures: Evidence From the Early Stages of the COVID-19 Pandemic
Source: Front Public Health. 2021 Jun 9;9:674597. doi: 10.3389/fpubh.2021.674597 (PMC8219917; doi:10.3389/fpubh.2021.674597)
Supplement: Supplementary file 1 [file Table_1.DOCX]

**Appendix**

| **Table A1**: Overview of sample size and prevalence (%) of adherence to preventive measures across countries | | | | | | | | | | | |
| --- | --- | --- | --- | --- | --- | --- | --- | --- | --- | --- | --- |
| Country |  | Wearing Masks  (prevalence, %) | | Keeping Distance  (prevalence, %) | | Washing Hands  (prevalence, %) | | Sanitizing Hands  (prevalence, %) | | Covering Coughs/  Sneezes  (prevalence, %) | |
|  | n | Unw. | Wgt | Unw. | Wgt | Unw. | Wgt | Unw. | Wgt | Unw. | Wgt |
| Germany | 1,497 | 93.19 | 91.2 | 96.33 | 95.99 | 86.77 | 84.85 | 72.61 | 72.72 | 90.18 | 89.69 |
| Sweden | 744 | 2.02 | 1.62 | 95.16 | 95.08 | 89.92 | 90.72 | 78.23 | 79.24 | 82.12 | 84.77 |
| Spain | 469 | 96.38 | 95.82 | 98.08 | 97.56 | 97.87 | 96.63 | 94.03 | 94.32 | 91.04 | 90.68 |
| Italy | 705 | 98.72 | 99.29 | 98.44 | 98.52 | 94.33 | 94.72 | 91.49 | 94.18 | 90.07 | 92.66 |
| France | 1,130 | 87.17 | 86.65 | 94.96 | 95.67 | 85.75 | 85.97 | 85.75 | 87.47 | 87.88 | 89.59 |
| Denmark | 1,066 | 2.81 | 2.59 | 97.56 | 97.81 | 91.46 | 91.18 | 95.97 | 95.69 | 92.78 | 92.56 |
| Greece | 776 | 81.31 | 81.68 | 92.01 | 91.5 | 93.43 | 93 | 85.31 | 85.55 | 93.56 | 93.7 |
| Switzerland | 935 | 27.17 | 22.07 | 94.97 | 95.87 | 90.8 | 91.36 | 88.02 | 89.34 | 88.56 | 91.43 |
| Belgium | 1,134 | 63.23 | 64.26 | 97.71 | 97.14 | 91.8 | 91.15 | 83.16 | 85 | 84.92 | 84.18 |
| Israel | 263 | 93.54 | 92.14 | 93.54 | 92.83 | 90.87 | 93.2 | 67.68 | 71.55 | 78.71 | 80.07 |
| Czech Republic | 1,240 | 98.71 | 99.14 | 91.94 | 93.25 | 83.47 | 86.35 | 82.34 | 84.35 | 81.45 | 84.13 |
| Poland | 987 | 95.64 | 95.77 | 94.43 | 94.34 | 91.79 | 91.94 | 85.11 | 85.89 | 91.08 | 91.92 |
| Luxembourg | 375 | 98.67 | 97.74 | 99.47 | 98.78 | 86.93 | 85.61 | 89.87 | 92.13 | 89.87 | 88.93 |
| Hungary | 154 | 88.96 | 94.99 | 95.45 | 97.34 | 85.71 | 83.47 | 76.62 | 74.2 | 97.4 | 95.33 |
| Slovenia | 1,193 | 91.87 | 92.38 | 95.56 | 96.02 | 88.01 | 88.71 | 92.96 | 93.53 | 73.51 | 74 |
| Estonia | 1,362 | 31.13 | 28.92 | 93.1 | 92.29 | 91.78 | 92.33 | 89.79 | 91.82 | 73.86 | 76.8 |
| Croatia | 460 | 60.65 | 57.64 | 87.39 | 85.68 | 89.78 | 89.26 | 88.48 | 89.11 | 90.87 | 89.24 |
| Lithuania | 625 | 96.16 | 95.98 | 96 | 95.92 | 94.24 | 94.37 | 88.32 | 89.46 | 86.88 | 86.45 |
| Bulgaria | 328 | 92.38 | 91.4 | 91.46 | 91.26 | 91.46 | 92.86 | 71.65 | 73.39 | 85.67 | 86.36 |
| Cyprus | 135 | 77.78 | 81.68 | 91.85 | 93.88 | 94.07 | 93.71 | 97.04 | 98.94 | 65.93 | 66.63 |
| Finland | 616 | 4.71 | 4.47 | 92.05 | 94.06 | 92.37 | 92.17 | 88.15 | 89.82 | 89.61 | 90.09 |
| Latvia | 398 | 24.87 | 26.34 | 88.44 | 87.48 | 89.95 | 89.19 | 82.91 | 80.78 | 84.17 | 84.42 |
| Romania | 542 | 91.51 | 92.21 | 96.49 | 97.01 | 92.44 | 91.34 | 79.7 | 80.46 | 89.11 | 88.67 |
| Slovakia | 454 | 95.81 | 96.04 | 84.36 | 84.68 | 86.56 | 86.81 | 82.16 | 82.05 | 70.93 | 71.16 |
| Total | 17,588 | 68.02 | 67.75 | 94.52 | 94.59 | 90.06 | 90.13 | 85.31 | 86.40 | 85.46 | 86.23 |
| Notes: For each measure, unweighted (Unw.) and weighted (Wgt.) prevalence rates are presented | | | | | | | | | | | |

| **Table A2**: Overview of sample size and prevalence (%) of adherence to preventive measures across countries | | | | | | | |
| --- | --- | --- | --- | --- | --- | --- | --- |
| Country |  | Went shopping since the start of pandemic : less often  (prevalence, %) | | Met more than 5 people since the start of the pandemic: less often  (prevalence, %) | | Visited people since the start of the pandemic less often  (prevalence, %) | |
|  | n | Unw. | Wgt. | Unw. | Wgt. | Unw. | Wgt. |
| Germany | 1,497 | 54.58 | 52.47 | 88.84 | 87.26 | 74.42 | 72.2 |
| Sweden | 744 | 71.77 | 62.53 | 82.93 | 77.09 | 81.45 | 75.42 |
| Spain | 469 | 85.93 | 79.84 | 95.52 | 96.67 | 93.82 | 92.28 |
| Italy | 705 | 81.28 | 82.48 | 95.74 | 95.12 | 92.06 | 90.9 |
| France | 1,130 | 70.62 | 69.43 | 90.18 | 90.52 | 82.48 | 83.06 |
| Denmark | 1,066 | 48.97 | 45.57 | 82.83 | 82.62 | 76.36 | 74.46 |
| Greece | 776 | 61.98 | 59.37 | 86.73 | 87.05 | 78.48 | 75.7 |
| Switzerland | 935 | 75.40 | 69.71 | 95.08 | 94.63 | 85.45 | 83.6 |
| Belgium | 1,134 | 72.57 | 69.33 | 95.50 | 93.95 | 89.51 | 86.28 |
| Israel | 263 | 66.54 | 60.94 | 86.69 | 85.13 | 85.55 | 80.25 |
| Czech Republic | 1,240 | 59.68 | 59.91 | 86.77 | 86.29 | 74.19 | 76.17 |
| Poland | 987 | 66.87 | 66.79 | 86.42 | 85.11 | 83.28 | 83.06 |
| Luxembourg | 375 | 82.13 | 80.54 | 97.33 | 97.3 | 90.13 | 86.29 |
| Hungary | 154 | 70.78 | 66.17 | 79.22 | 71.36 | 86.36 | 87.9 |
| Slovenia | 1,193 | 88.52 | 86.95 | 95.31 | 94.3 | 92.88 | 92.77 |
| Estonia | 1,362 | 72.61 | 70.44 | 80.10 | 76.17 | 86.93 | 84.87 |
| Croatia | 460 | 68.48 | 64.97 | 90.43 | 86.9 | 85.87 | 84.36 |
| Lithuania | 625 | 80.80 | 79.23 | 89.28 | 86.96 | 89.60 | 88.28 |
| Bulgaria | 328 | 46.95 | 44.29 | 69.21 | 66.23 | 64.63 | 64 |
| Cyprus | 135 | 78.52 | 80.77 | 91.85 | 89.47 | 90.37 | 84.93 |
| Finland | 616 | 65.58 | 63.49 | 85.39 | 85.38 | 80.52 | 81.67 |
| Latvia | 398 | 64.57 | 61.6 | 84.42 | 81.85 | 86.18 | 83.37 |
| Romania | 542 | 80.44 | 79.16 | 92.07 | 88.99 | 89.30 | 88.15 |
| Slovakia | 454 | 45.15 | 48.12 | 70.93 | 71.71 | 68.94 | 69.18 |
| Total | 17,588 | 68.65 | 66.45 | 88.11 | 86.71 | 83.20 | 81.86 |
| Notes: For each measure, unweighted (Unw.) and weighted (Wgt.) prevalence rates are presented. | | | | | | | |

| **Table A3**: Overview of sample size and prevalence (%) of behavioural risk factors across countries | | | | | | | | | | | |
| --- | --- | --- | --- | --- | --- | --- | --- | --- | --- | --- | --- |
| Country |  | Overweight  (prevalence, %) | | Presently smoking  (prevalence, %) | | No daily fruits and veg.  (prevalence, %) | | Physical inactivity  (prevalence, %) | | Risky alcohol consumption  (prevalence, %) | |
|  | n | Unw. | Wgt. | Unw. | Wgt. | Unw. | Wgt. | Unw. | Wgt. | Unw. | Wgt. |
| Germany | 1,497 | 23.71 | 24.73 | 16.30 | 20.55 | 27.32 | 32.47 | 43.55 | 40.91 | 9.89 | 10.23 |
| Sweden | 744 | 17.88 | 17.53 | 5.78 | 7.25 | 21.64 | 22.32 | 45.16 | 40.24 | 4.97 | 6.1 |
| Spain | 469 | 27.08 | 25.59 | 11.94 | 20.38 | 14.93 | 17.54 | 63.75 | 60.63 | 3.62 | 2.03 |
| Italy | 705 | 14.75 | 12.56 | 16.45 | 20.08 | 11.21 | 12.34 | 57.16 | 55.46 | 6.52 | 6.18 |
| France | 1,130 | 19.73 | 19.49 | 10.97 | 14.76 | 10.44 | 13.11 | 59.38 | 53.35 | 2.57 | 4.23 |
| Denmark | 1,066 | 17.92 | 18.24 | 14.73 | 16.70 | 27.02 | 27.48 | 35.65 | 34.73 | 4.03 | 5.04 |
| Greece | 776 | 21.39 | 21.45 | 22.29 | 26.48 | 37.24 | 36.95 | 57.99 | 55.11 | 4.77 | 6.51 |
| Switzerland | 935 | 14.33 | 16.00 | 12.62 | 16.11 | 16.04 | 20.48 | 40.21 | 40.61 | 12.09 | 10.88 |
| Belgium | 1,134 | 20.11 | 19.79 | 12.08 | 12.70 | 13.05 | 13.42 | 58.11 | 51.18 | 9.52 | 9.51 |
| Israel | 263 | 17.87 | 15.01 | 12.17 | 14.17 | 22.05 | 20.56 | 44.49 | 44.93 | 1.52 | 1.67 |
| Czech Republic | 1,240 | 32.18 | 34.77 | 17.58 | 18.73 | 26.77 | 27.43 | 60.32 | 60.72 | 5.24 | 4.44 |
| Poland | 987 | 29.89 | 29.59 | 23.81 | 27.13 | 32.83 | 36.94 | 64.84 | 62.63 | 2.03 | 3.07 |
| Luxembourg | 375 | 22.13 | 21.49 | 14.13 | 12.92 | 13.33 | 12.25 | 45.87 | 42.8 | 10.40 | 11.25 |
| Hungary | 154 | 32.47 | 38.63 | 20.13 | 24.34 | 50.65 | 46.31 | 64.29 | 73.11 | 8.44 | 8.59 |
| Slovenia | 1,193 | 26.74 | 27.36 | 12.24 | 18.51 | 12.91 | 13.68 | 40.82 | 37.58 | 3.35 | 2.94 |
| Estonia | 1,362 | 33.48 | 35.13 | 15.93 | 21.18 | 18.87 | 19.72 | 56.39 | 50.79 | 6.09 | 8.29 |
| Croatia | 460 | 28.48 | 29.32 | 25.43 | 28.85 | 27.39 | 29.68 | 50.87 | 49.04 | 8.48 | 8.09 |
| Lithuania | 625 | 30.24 | 29.75 | 14.88 | 17.86 | 29.12 | 29.05 | 53.92 | 49.52 | 3.36 | 4.41 |
| Bulgaria | 328 | 26.83 | 28.84 | 18.90 | 22.76 | 66.16 | 65.87 | 56.71 | 54.2 | 15.55 | 19.49 |
| Cyprus | 135 | 23.70 | 26.37 | 12.59 | 23.75 | 28.89 | 30 | 64.44 | 68.4 | 21.48 | 19.49 |
| Finland | 616 | 24.03 | 25.46 | 10.23 | 9.94 | 22.56 | 26.65 | 33.77 | 30.45 | 6.17 | 5.76 |
| Latvia | 398 | 38.19 | 38.78 | 11.06 | 13.55 | 30.90 | 31.95 | 39.20 | 35.36 | 5.28 | 6.7 |
| Romania | 542 | 31.73 | 31.36 | 16.24 | 15.53 | 54.80 | 58.04 | 45.20 | 39.39 | 10.89 | 12.55 |
| Slovakia | 454 | 24.67 | 23.79 | 18.06 | 17.78 | 58.15 | 58.11 | 51.76 | 53.05 | 10.35 | 9.74 |
| Total | 17,588 | 24.64 | 25.13 | 15.16 | 18.25 | 24.74 | 26.37 | 50.86 | 48.07 | 6.52 | 7.06 |
| Notes: For each behavioral risk factor, unweighted (Unw.) and weighted (Wgt.) prevalence rates are presented. Prevalence of overweight and obese BMIs, compared to normal weight and underweight. Unhealthy eating is defined as no daily intake of fruits and vegetables. Physical inactivity characterizes less than weekly physical activity. Risky alcohol consumption is defined as drinking 6+ units of alcohol on one occasion at least weekly in the last three months. | | | | | | | | | | | |

| **Table A4:** Multilevel analyses predicting the preventive measures including disaggregated behavioral risk factors (n = 17,588), Data: Wave 8 Release 0.0.1 beta | | | | | | | | | | |
| --- | --- | --- | --- | --- | --- | --- | --- | --- | --- | --- |
|  | |  | (1) | | | | (2) | | (3) | |
|  | |  | Social Isolation | | | | Hygiene Measures | | Regulated Measures | |
| Regressors | | | |  | OR | CI (95%) | OR | CI (95%) | OR | CI (95%) |
|  | | |  | | |  |  |  |  |  |
| BRFs | | |  | | |  |  |  |  |  |
|  | BMI >= 25 | | 1.03 | | | [0.93, 1.15] | 0.91 | [0.75,1.09] | 0.93 | [0.78, 1.10] |
|  | Presently smoking | | 0.73*** | | | [0.62, 0.86] | 0.87** | [0.79,0.97] | 0.89 | [0.78, 1.03] |
|  | No daily fruits and vegetables | | 1.14*** | | | [1.05, 1.25] | 0.97 | [0.78,1.21] | 0.75** | [0.56, 0.99] |
|  | Physical inactivity | | 1.08 | | | [0.97, 1.20] | 0.91 | [0.78,1.07] | 1.00 | [0.86, 1.17] |
|  | Risky alcohol consumption | | 1.05 | | | [0.85, 1.29] | 0.91 | [0.79,1.05] | 0.90 | [0.64, 1.26] |
| Sex | | |  | | |  |  |  |  |  |
|  | male (*Ref*.) | |  | | |  |  |  |  |  |
|  | female | | 1.72*** | | | [1.25, 2.35] | 1.41*** | [1.28,1.55] | 1.53*** | [1.31,1.79] |
| Age | | |  | | |  |  |  |  |  |
|  | 50-55 y/o | | 1.03 | | | [0.71, 1.49] | 1.32*** | [1.07,1.62] | 1.51*** | [1.17, 1.94] |
|  | 56-64 y/o (*Ref.*) | |  | | |  |  |  |  |  |
|  | 65-79 y/o | | 1.21* | | | [0.98, 1.49] | 0.87* | [0.73,1.02] | 0.98 | [0.65, 1.50] |
|  | Older than 80 y/o | | 1.40*** | | | [1.17, 1.68] | 0.65*** | [0.52,0.80] | 0.64* | [0.40,1.03] |
| Living situation | | |  | | |  |  |  |  |  |
|  | Living alone (*Ref.*) | |  | | |  |  |  |  |  |
|  | Spouse/partner in HH | | 1.44** | | | [1.09, 1.89] | 1.46*** | [1.34,1.59] | 1.18 | [0.95,1.47] |
| Education | | |  | | |  |  |  |  |  |
|  | Primary (*Ref.*) | |  | | |  |  |  |  |  |
|  | Secondary | | 1.02 | | | [0.95, 1.09] | 1.24** | [1.01,1.53] | 1.14 | [0.85, 1.53] |
|  | Tertiary or above | | 1.10 | | | [0.90, 1.34] | 1.71*** | [1.48,1.97] | 1.43 | [0.90, 2.29] |
| Employment | | |  | | |  |  |  |  |  |
|  | Retired (*Ref.*) | |  | | |  |  |  |  |  |
|  | Employed/Self-employed | | 0.91 | | | [0.70, 1.18] | 1.26** | [1.02,1.57] | 0.83 | [0.62, 1.10] |
|  | Other | | 0.98 | | | [0.85, 1.14] | 1.12 | [0.87,1.44] | 0.88 | [0.73, 1.06] |
| Living environment | | |  | | |  |  |  |  |  |
|  | Rural (*Ref*.) | |  | | |  |  |  |  |  |
|  | Urban | | 0.94 | | | [0.81, 1.10] | 1.07 | [0.87,1.31] | 1.20 | [0.95, 1.50] |
| Depression risk (EURO-D) | | | 1.05 | | | [0.97, 1.14] | 0.96 | [0.87,1.06] | 1.03 | [0.90, 1.16] |
| Subjective health | | |  | | |  |  |  |  |  |
|  | Fair/poor (*Ref*.) | |  | | |  |  |  |  |  |
|  | At least good | | 0.59*** | | | [0.44, 0.79] | 1.00 | [0.90,1.12] | 1.12 | [0.86,1.46] |
| No. of chronic diseases | | | 1.03 | | | [0.95, 1.12] | 1.07** | [1.01,1.14] | 1.18*** | [1.04, 1.33] |
| Big Five personality traits | | |  | | |  |  |  |  |  |
|  | Openness | | 1.01 | | | [0.98, 1.03] | 0.98** | [0.96,1.00] | 1.04** | [1.00, 1.09] |
|  | Conscientiousness | | 0.99 | | | [0.95, 1.04] | 1.05** | [1.01, 1.09] | 1.15*** | [1.06,1.24] |
|  | Extraversion | | 0.98* | | | [0.95, 1.00] | 0.96*** | [0.93, 0.99] | 0.95*** | [0.91,0.98] |
|  | Agreeableness | | 1.01 | | | [0.97, 1.05] | 1.08*** | [1.06, 1.11] | 1.05 | [0.98,1.11] |
|  | Neuroticism | | 1.04** | | | [1.00, 1.07] | 1.03** | [1.00, 1.05] | 1.04** | [1.01,1.08] |
| ICC |  | | 0.09 | | |  | 0.09 |  | 0.07 |  |
| BIC |  | | 23226.51 | | |  | 22601.45 |  | 22601.28 |  |
| AIC | | | |  | 23210.96 |  | 22422.62 |  | 22414.68 |  |
| Note: OR Odds ratios, CI (95%) = confidence interval 95%, Var. = variance component, *** p<0.01, ** p<0.05, * p<0.1 | | | | | | | | | | |

| **Table A5:** Multilevel analyses predicting the preventive measures including disaggregated outcomes and BRFs (n = 17,588), Data: Wave 8 Release 0.0.1 beta | | | | | | | | | | |  |  |
| --- | --- | --- | --- | --- | --- | --- | --- | --- | --- | --- | --- | --- |
|  | |  | | | (1) | | (2) | | (3) | | (4) | |
|  | |  | | | Wore mask | | Kept distance | | Washed hands | | Hand Sanitizing | |
| Regressors | | |  | OR | | CI (95%) | OR |  | CI (95%) | CI (95%) | OR | CI (95%) |
| BRFs | | | | |  |  |  |  |  |  |  |  |
|  | BMI >= 25 | | | | 0.91 | [0.75,1.11] | 0.97 | [0.75,1.26] | 1.02 | [0.88,1.20] | 0.92 | [0.80,1.06] |
|  | Presently smoking | | | | 0.91 | [0.73,1.12] | 0.91 | [0.66,1.24] | 0.74*** | [0.67,0.81] | 0.80*** | [0.68,0.93] |
|  | Unhealthy food intake | | | | 0.82** | [0.69,0.97] | 0.71 | [0.45,1.10] | 1.13* | [0.99,1.29] | 1.1 | [0.83,1.45] |
|  | Physical inactivity | | | | 1.06 | [0.89,1.26] | 0.78* | [0.59,1.03] | 0.82* | [0.67,1.01] | 0.81** | [0.69,0.96] |
|  | Risky alcohol consumption | | | | 0.95 | [0.55,1.62] | 0.60*** | [0.42,0.86] | 0.78*** | [0.65,0.93] | 0.86 | [0.63,1.17] |
| Sex | | | | |  |  |  |  |  |  |  |  |
|  | male (*Ref*.) | | | |  |  |  |  |  |  |  |  |
|  | female | | | | 1.50*** | [1.32,1.71] | 1.63*** | [1.24,2.15] | 1.31*** | [1.16,1.48] | 1.36*** | [1.10,1.70] |
| Age | | | | |  |  |  |  |  |  |  |  |
|  | 50-55 y/o | | | | 1.62*** | [1.24,2.12] | 1.48 | [0.87,2.54] | 1.38 | [0.84,2.25] | 0.98 | [0.60,1.60] |
|  | 56-64 y/o (*Ref.*) | | | |  |  |  |  |  |  |  |  |
|  | 65-79 y/o | | | | 1.06 | [0.73,1.53] | 0.92 | [0.76,1.11] | 1.23* | [0.98,1.55] | 0.76*** | [0.65,0.89] |
|  | Older than 80 y/o | | | | 0.68 | [0.40,1.16] | 0.48*** | [0.38,0.61] | 1.01 | [0.75,1.37] | 0.49*** | [0.38,0.63] |
| Living situation | | | | |  |  |  |  |  |  |  |  |
|  | Living alone (*Ref.*) | | | |  |  |  |  |  |  |  |  |
|  | Spouse/partner in HH | | | | 1.13 | [0.89,1.43] | 1.3 | [0.85,1.99] | 1.32*** | [1.12,1.57] | 1.60*** | [1.42,1.79] |
| Education | | | | |  |  |  |  |  |  |  |  |
|  | Primary (*Ref.*) | | | |  |  |  |  |  |  |  |  |
|  | Secondary | | | | 1.29* | [0.96,1.74] | 1.21 | [0.85,1.73] | 1.12 | [0.87,1.44] | 1.51*** | [1.25,1.83] |
|  | Tertiary or above | | | | 1.62*** | [1.16,2.25] | 1.55 | [0.81,2.96] | 1.32** | [1.01,1.74] | 1.88*** | [1.41,2.52] |
| Employment | | | | |  |  |  |  |  |  |  |  |
|  | Retired (*Ref.*) | | | |  |  |  |  |  |  |  |  |
|  | Employed/Self-employed | | | | 0.83* | [0.68,1.02] | 0.9 | [0.63,1.29] | 1.31*** | [1.07,1.59] | 1.26* | [0.97,1.65] |
|  | Other | | | | 0.83 | [0.66,1.05] | 1.33* | [0.98,1.80] | 1.01 | [0.85,1.21] | 1.04 | [0.83,1.32] |
| Living environment | | | | |  |  |  |  |  |  |  |  |
|  | Rural (*Ref*.) | | | |  |  |  |  |  |  |  |  |
|  | Urban | | | | 1.17 | [0.81,1.69] | 1.36** | [1.02,1.80] | 1.32*** | [1.12,1.56] | 1.22* | [0.97,1.54] |
| Depression risk (EURO-D) | | | | | 1.11 | [0.95,1.29] | 0.97 | [0.81,1.17] | 1.02 | [0.93,1.12] | 1 | [0.93,1.08] |
| Subjective health | | | | |  |  |  |  |  |  |  |  |
|  | Fair/poor (*Ref*.) | | | |  |  |  |  |  |  |  |  |
|  | At least good | | | | 1.28 | [0.94,1.75] | 0.85 | [0.59,1.22] | 0.94 | [0.62,1.41] | 1 | [0.84,1.18] |
| No. of chronic diseases | | | | | 1.21*** | [1.06,1.39] | 1.08 | [0.91,1.27] | 1.04** | [1.00,1.07] | 1.10*** | [1.05,1.15] |
| Big Five personality traits | | | | |  |  |  |  |  |  |  |  |
|  | Openness | | | | 1.02 | [0.98,1.06] | 1.11*** | [1.04,1.18] | 1.01 | [0.96,1.07] | 0.96** | [0.93,0.99] |
|  | Conscientiousness | | | | 1.19*** | [1.11,1.27] | 1.06 | [0.97,1.15] | 1.03 | [0.99,1.07] | 1.03 | [0.99,1.08] |
|  | Extraversion | | | | 0.97 | [0.94,1.01] | 0.87*** | [0.79,0.96] | 1.02 | [0.97,1.06] | 0.94*** | [0.90,0.98] |
|  | Agreeableness | | | | 1.06 | [0.98,1.15] | 1.02 | [0.94,1.11] | 1.08*** | [1.05,1.11] | 1.07*** | [1.04,1.10] |
|  | Neuroticism | | | | 1.07*** | [1.02,1.12] | 0.98 | [0.91,1.06] | 1.05** | [1.00,1.10] | 1 | [0.96,1.04] |
| ICC |  | | | | 0.56 |  | 0.08 |  | 0.08 |  | 0.15 |  |
| BIC |  | | | | 9581.87 |  | 6085.78 |  | 11834.77 |  | 14640.40 |  |
| AIC | | |  | 9403.04 | |  | 5906.96 |  | 11655.95 |  | 14461.58 |  |
| Note: OR Odds ratios, CI (95%) = confidence interval 95%, Var. = variance component, *** p<0.01, ** p<0.05, * p<0.1 | | | | | | | | | | |  |  |

| **Table A5:** *(continued)* Multilevel analyses predicting the preventive measures including disaggregated outcomes and BRFs (n = 17,588), Data: Wave 8 Release 0.0.1 beta | | | | | | | | | | |  |  |
| --- | --- | --- | --- | --- | --- | --- | --- | --- | --- | --- | --- | --- |
|  | |  | | | (5) | | (6) | | (7) | | (8) | |
|  | |  | | | Covered coughs/sneezes | | Visited people | | Met more than 5 people | | Went shopping | |
| Regressors | | |  | OR | | CI (95%) | OR |  | CI (95%) | CI (95%) | OR | CI (95%) |
| BRFs | | | | |  |  |  |  |  |  |  |  |
|  | BMI >= 25 | | | | 0.76* | [0.56,1.04] | 1.09** | [1.01,1.18] | 0.99 | [0.83,1.18] | 0.95 | [0.85,1.08] |
|  | Presently smoking | | | | 1.12 | [0.82,1.53] | 1.06 | [0.96,1.16] | 0.94 | [0.80,1.11] | 0.72*** | [0.62,0.82] |
|  | Unhealthy food intake | | | | 0.85* | [0.71,1.01] | 1.07 | [0.97,1.18] | 1.07 | [0.87,1.32] | 1.05 | [0.93,1.19] |
|  | Physical inactivity | | | | 0.92 | [0.71,1.20] | 1.05 | [0.95,1.15] | 1.09 | [0.93,1.28] | 1.07* | [0.99,1.17] |
|  | Risky alcohol consumption | | | | 1.14 | [0.87,1.49] | 0.89* | [0.77,1.02] | 0.65*** | [0.58,0.72] | 1.08 | [0.89,1.31] |
| Sex | | | | |  |  |  |  |  |  |  |  |
|  | male (*Ref*.) | | | |  |  |  |  |  |  |  |  |
|  | female | | | | 1.58*** | [1.36,1.83] | 1.58*** | [1.15,2.18] | 1.34 | [0.90,2.00] | 1.89*** | [1.46,2.45] |
| Age | | | | |  |  |  |  |  |  |  |  |
|  | 50-55 y/o | | | | 1.70* | [0.97,2.98] | 0.91 | [0.72,1.15] | 0.94 | [0.64,1.40] | 0.96 | [0.68,1.34] |
|  | 56-64 y/o (*Ref.*) | | | |  |  |  |  |  |  |  |  |
|  | 65-79 y/o | | | | 0.72** | [0.54,0.97] | 1.16 | [0.91,1.48] | 1.11 | [0.89,1.40] | 1.11 | [0.92,1.35] |
|  | Older than 80 y/o | | | | 0.53*** | [0.37,0.75] | 1.07 | [0.81,1.42] | 1.33 | [0.90,1.97] | 1.28** | [1.05,1.55] |
| Living situation | | | | |  |  |  |  |  |  |  |  |
|  | Living alone (*Ref.*) | | | |  |  |  |  |  |  |  |  |
|  | Spouse/partner in HH | | | | 1.32*** | [1.18,1.46] | 1.51*** | [1.12,2.04] | 1.30*** | [1.09,1.57] | 1.40** | [1.06,1.85] |
| Education | | | | |  |  |  |  |  |  |  |  |
|  | Primary (*Ref.*) | | | |  |  |  |  |  |  |  |  |
|  | Secondary | | | | 1.48*** | [1.15,1.90] | 0.93 | [0.72,1.20] | 1.21 | [0.75,1.94] | 1.05 | [0.91,1.21] |
|  | Tertiary or above | | | | 1.74*** | [1.34,2.27] | 1.05 | [0.76,1.44] | 1.4 | [0.92,2.15] | 1.09 | [0.89,1.34] |
| Employment | | | | |  |  |  |  |  |  |  |  |
|  | Retired (*Ref.*) | | | |  |  |  |  |  |  |  |  |
|  | Employed/Self-employed | | | | 1.08 | [0.79,1.47] | 1.04 | [0.74,1.47] | 0.75 | [0.49,1.15] | 0.88 | [0.71,1.10] |
|  | Other | | | | 0.91 | [0.63,1.32] | 0.83*** | [0.73,0.95] | 0.87 | [0.64,1.18] | 0.89 | [0.71,1.11] |
| Living environment | | | | |  |  |  |  |  |  |  |  |
|  | Rural (*Ref*.) | | | |  |  |  |  |  |  |  |  |
|  | Urban | | | | 0.81** | [0.66,0.98] | 1.12 | [0.95,1.33] | 1.28*** | [1.12,1.47] | 0.91 | [0.71,1.17] |
| Depression risk (EURO-D) | | | | | 1.1 | [0.78,1.54] | 0.96 | [0.82,1.12] | 0.93 | [0.77,1.13] | 1.06 | [0.96,1.17] |
| Subjective health | | | | |  |  |  |  |  |  |  |  |
|  | Fair/poor (*Ref*.) | | | |  |  |  |  |  |  |  |  |
|  | At least good | | | | 1.02 | [0.86,1.20] | 0.54*** | [0.40,0.73] | 0.57*** | [0.41,0.80] | 0.66*** | [0.51,0.85] |
| No. of chronic diseases | | | | | 1.09** | [1.02,1.17] | 1.06* | [0.99,1.13] | 1 | [0.93,1.07] | 1.02 | [0.95,1.09] |
| Big Five personality traits | | | | |  |  |  |  |  |  |  |  |
|  | Openness | | | | 0.98 | [0.93,1.03] | 1.01 | [0.99,1.04] | 0.99 | [0.95,1.03] | 1.02 | [0.99,1.05] |
|  | Conscientiousness | | | | 1.09** | [1.01,1.18] | 0.99 | [0.95,1.02] | 0.98 | [0.95,1.02] | 1.01 | [0.99,1.04] |
|  | Extraversion | | | | 0.95** | [0.91,1.00] | 0.99 | [0.96,1.02] | 0.98 | [0.95,1.02] | 0.97** | [0.94,1.00] |
|  | Agreeableness | | | | 1.11*** | [1.03,1.18] | 0.96*** | [0.94,0.99] | 1 | [0.98,1.03] | 1.02 | [0.98,1.07] |
|  | Neuroticism | | | | 1.05 | [0.99,1.11] | 1.01 | [0.97,1.05] | 1.07*** | [1.02,1.12] | 1.04** | [1.00,1.08] |
| ICC |  | | | | 0.05 |  | 0.09 |  | 0.16 |  | 0.09 |  |
| BIC |  | | | | 11607.62 |  | 16429.24 |  | 12032.78 |  | 21365.98 |  |
| AIC | | |  | 11428.79 | |  | 16250.41 |  | 11853.95 |  | 21187.15 |  |
| Note: OR Odds ratios, CI (95%) = confidence interval 95%, Var. = variance component, *** p<0.01, ** p<0.05, * p<0.1 | | | | | | | | | | |  |  |

| **Table A6:** Analyses predicting the preventive measures using alternative models (n = 17,588), Data: Wave 8 Release 0.0.1 beta | | | | | | | | |
| --- | --- | --- | --- | --- | --- | --- | --- | --- |
|  |  | | (1) | | (2) | | (3) | |
|  |  | | Social Isolation | | Hygiene Measures | | Regulated Measures | |
|  |  | |  | |  | |  | |
| *Panel A: Multi-level logistic regression (weighted)* | | | OR | CI (95%) | OR | CI (95%) | OR | CI (95%) |
| BRFs | | 1 BRF | 1.09 | [0.94,1.27] | 0.85*** | [0.78,0.94] | 0.98 | [0.67,1.42] |
|  | | 2 BRFs | 1.01 | [0.82,1.25] | 0.85** | [0.75,0.96] | 1.06 | [0.77,1.46] |
|  | | 3+ BRFs | 1.06 | [0.95,1.18] | 0.72*** | [0.59,0.88] | 0.84 | [0.63,1.13] |
|  | |  |  |  |  |  |  |  |
| *Panel B: Multi-level logistic regression (unweighted)* | | | OR | CI (95%) | OR | CI (95%) | OR | CI (95%) |
| BRFs | | 1 BRF | 0.95 | [0.88,1.03] | 1.01 | [0.92,1.11] | 1.09 | [0.97,1.24] |
|  | | 2 BRFs | 0.96 | [0.88,1.05] | 0.94 | [0.85,1.04] | 1.04 | [0.95,1.14] |
|  | | 3+ BRFs | 0.91** | [0.83,1.00] | 0.86*** | [0.77,0.95] | 0.99 | [0.86,1.13] |
|  | |  |  |  |  |  |  |  |
| *Panel C: Logistic regression with country fixed effects (weighted)* | | | OR | CI (95%) | OR | CI (95%) | OR | CI (95%) |
| BRFs | | 1 BRF | 1.12 | [0.92,1.37] | 0.85*** | [0.76,0.94] | 0.97 | [0.66,1.44] |
|  | | 2 BRFs | 0.97 | [0.72,1.30] | 0.83** | [0.69,1.00] | 1.05 | [0.75,1.47] |
|  | | 3+ BRFs | 1.09 | [0.89,1.34] | 0.74*** | [0.59,0.92] | 0.84 | [0.65,1.09] |
|  | |  |  |  |  |  |  |  |
| *Panel D: Multi-level linear regression (weighted)* | | | β | CI (95%) | β | CI (95%) | β | CI (95%) |
| BRFs | | 1 BRF | 0.02 | [-0.01,0.05] | -0.03*** | [-0.04,-0.01] | 0 | [-0.03,0.03] |
|  | | 2 BRFs | 0 | [-0.04,0.05] | -0.03*** | [-0.05,-0.01] | 0.01 | [-0.02,0.04] |
|  | | 3+ BRFs | 0.01 | [-0.01,0.04] | -0.06*** | [-0.10,-0.03] | -0.01 | [-0.04,0.01] |
|  | |  |  |  |  |  |  |  |
| Notes: All regressions include a set of for socio-demographic and health variables as controls. The reference category of the BRFs index is “0 Behavioral Risk Factors”. OR = Odds ratios, CI (95%) = confidence interval 95%, *** p<0.01, ** p<0.05, * p<0.1 | | | | | | | | |
